# Unique features of microglial response in Progressive Supranuclear Palsy

**DOI:** 10.1002/alz70855_103280

**Published:** 2025-12-24

**Authors:** Satoshi Tanikawa, Gabor G. Kovacs

**Affiliations:** ^1^ Tanz Centre for Research in Neurodegenerative Diseases, Toronto, ON, Canada; ^2^ Department of Laboratory Medicine and Pathobiology, University of Toronto, Toronto, ON, Canada; ^3^ Tanz Centre for Research in Neurodegenerative Diseases, University of Toronto, Toronto, ON, Canada; ^4^ Laboratory Medicine Program, University Health Network, Toronto, ON, Canada; ^5^ Rossy PSP Program, University Health Network and the University of Toronto, Toronto, ON, Canada; ^6^ The Edmond J. Safra Program in Parkinson's Disease and Morton and Gloria Shulman Movement Disorders Clinic, Toronto, ON, Canada; ^7^ Krembil Brain Institute, Toronto, ON, Canada

## Abstract

**Background:**

Microglia play a crucial role in neurodegenerative diseases, but their specific involvement in disease progression remains unclear. Microglial diversity, initially described by Río‐Hortega as varied morphological features, has been further explored through single‐cell analyses identifying multiple subtypes. These findings suggest microglia exhibit diverse morphological and functional changes depending on pathological conditions even within the same disease. However, there is a paucity of data on progressive supranuclear palsy a (PSP), a tauopathy characterized by neuronal and glial tau pathologies.

**Method:**

This study investigates the role of microglia in the frontal cortex of PSP. We performed immunohistochemistry for Iba1 and HLA‐DR microglial markers in nine PSP cases and nine age‐matched controls. Quantitative evaluation of positive regions in the cortex and white matter was conducted using HALO®. Morphological analysis of Iba1‐positive cells was performed with the HALO Microglial Activation Module. The snRNA‐seq on frozen samples from three PSP and three controls identified differentially expressed genes (DEGs) and GO enrichment. PSP‐specific microglial subclusters were compared with previously reported subtypes (DAM, MgND, HAM).

**Result:**

Iba1‐positive microglia load is significantly increased only in the white matter in PSP, while HLA‐DR‐positive microglia load is increased in both the cortex and white matter. Microglia in PSP exhibited significantly longer processes, more branching, and larger cell bodies, particularly in the white matter. The snRNA‐seq identified four PSP‐specific subclusters, including those with high homeostatic gene expression (SC0), MHC class II pathway activation (SC0, 2, 4) and clusters enriched with aging‐related genes (SC5). GO enrichment revealed functional diversity, including clusters enriched with aging‐related genes. Modulation scoring did not reveal clear similarities with previously reported microglial subtype gene sets.

**Conclusion:**

This study reveals unique microglial morphological and functional characteristics in the PSP affected frontal cortex.

